# Agricultural systems in Bangladesh: the first archaeobotanical results from Early Historic Wari-Bateshwar and Early Medieval Vikrampura

**DOI:** 10.1007/s12520-019-00991-5

**Published:** 2020-01-15

**Authors:** Mizanur Rahman, Cristina Cobo Castillo, Charlene Murphy, Sufi Mostafizur Rahman, Dorian Q. Fuller

**Affiliations:** 1 Department of Archaeology, Faculty of Arts and Humanities, Jahangirnagar University, Savar, Dhaka 1342 Bangladesh; 2 School of Archaeology, University of Oxford, Oxford, OX1 2PG England; 3 UCL Institute of Archaeology, 31-34 Gordon Square, London, WC1H 0PY UK; 4 School of Cultural Heritage, Northwest University, Xi’an, Shaanxi China

**Keywords:** Palaeoethnobotany, South Asia, Southeast Asia, Oryza, Gossypium, *Vigna*, Sesamum, Brassica

## Abstract

The present paper reports the first systematic archaeobotanical evidence from Bangladesh together with direct AMS radiocarbon dates on crop remains. Macro-botanical remains were collected by flotation from two sites, Wari-Bateshwar (WB), an Early Historic archaeological site, dating mainly between 400 and 100 BC, with a later seventh century AD temple complex, and Raghurampura Vikrampura (RV), a Buddhist Monastery (*vihara*) located within the Vikrampura city site complex and dating to the eleventh and sixteenth centuries AD. Despite being a tropical country, with high rainfall and intensive soil processes, our work demonstrates that conventional archaeobotany, the collection of macro-remains through flotation, has much potential towards putting together a history of crops and agricultural systems in Bangladesh. The archaeobotanical assemblage collected from both sites indicates the predominance of rice agriculture, which would have been practiced in summer. Spikelet bases are of domesticated type rice, while grain metrics suggest the majority of rice was probably subspecies *japonica.* The presence of some wetland weeds suggests at least some of the rice was grown in wet (flooded) systems, but much of it may have been rainfed as inferred from the Southeast Asian weed *Acmella paniculata*. Other crops include winter cereals, barley and possible oat, and small numbers of summer millets (*Pennisetum glaucum*, *Sorghum bicolor*, *Setaria italica*), a wide diversity of summer and winter pulses (14 spp.), cotton, sesame and mustard seed. Pulse crops included many known from India. Thus, while most crops indicate diffusion of crops from India eastwards, the absence of *indica* rice could also indicate some diffusion from Southeast Asia. The later site RV also produced evidence of the rice bean (*Vigna umbellata*), a domesticate of mainland Southeast Asia. These data provide the first empirical evidence for reconstructing past agriculture in Bangladesh and for the role of connections to both India and mainland Southeast Asia in the development of crop diversity in the Ganges delta region.

**Electronic supplementary material:**

The online version of this article (10.1007/s12520-019-00991-5) contains supplementary material, which is available to authorized users.

## Introduction

Bangladesh lies in the geographical transition from South Asia (the Indian subcontinent) to mainland Southeast Asia. Despite advances in the archaeobotany of India (e.g. Fuller 2011; Pokharia et al. 2014, 2017; Petrie and Bates 2017), China (e.g. Zhao 2011; Stevens et al. 2016) and some parts of Southeast Asia (e.g. Castillo and Fuller 2010; Castillo 2011, 2017a), the interrelations between South and Southeast Asia over land are poorly understood, especially with regard to the development of agricultural systems. A recent synthesis suggests that despite similarities in environment and diversity of crop taxa, the South Asian and Southeast Asian prehistories of agriculture were largely distinct with small degrees of relatively late borrowing (Fuller et al. 2016a). The first evidence for South Asian crops, such as mung beans (*Vigna radiata*) and horsegram (*Macrotyloma uniflorum*), in mainland Southeast Asia dates from the final centuries BC and is associated with maritime trade (Castillo et al. 2016a), and although earlier overland crop diffusion from the west is possible, there is no evidence at present. Evidence of overland crop movements from China to Southeast Asia from the 3rd and 2nd millennium BC have been documented but the mechanisms are still poorly understood and may have included farmer migrations (Higham 2014; Castillo 2017a; Stevens and Fuller 2017). Current integration of archaeobotanical and genetic evidence suggests two distinct stories of early rice (*Oryza sativa*) development in China and India, but with an apparent hybridization between early East Asian subspecies *japonica* and proto-*indica* proposed to have taken place in the Indo-Gangetic alluvial areas starting around 1900 BC (Fuller et al. 2010; Fuller 2011; Gross and Zhao 2014; Stevens et al. 2016; Choi et al. 2017). While it has been proposed that early connections between China and India led to the transfer of crops via central Asia (Fuller and Boivin 2009; Fuller 2011; Stevens et al. 2016), one weakness of the archaeological evidence is the lack of an archaeobotanical record between eastern India and southwest China. Thus, some scholars, based primarily on linguistic inferences and to some extent, crop varietal diversity, have argued for a key region of origin and dispersal of rice to be in northeast India (Assam) or thereabouts, such as adjacent Bangladesh (e.g. Hazarika 2006; van Driem 2012). Another unresolved problem is the origins of *aus* rices, which recent genetic research indicates are at least as different from standard *indica* and *japonica* as those two subspecies are from each other (Garris et al. 2005; McNally et al. 2009; Schwatz et al. 2014; Choi et al. 2017), and this suggests a separate origin. The core *aus* diversity is to be found in northeast India and adjacent Bangladesh and to a lesser degree parts of Myanmar (Travis et al. 2015; cf. Hossain et al. 2012). Thus, the regions at the junction between South and Southeast Asia are key to unravelling the extent of independent agricultural origins in different parts of tropical Asia as well as early interconnections and crop dispersal.

The present paper reports the first systematic archaeobotanical evidence from Bangladesh, collected by flotation from two sites, and uses this evidence to argue for an early (late 1st millennium BC) agricultural dispersal eastwards from India with diffusion from mainland Southeast Asia happening during the Historic period. Thus, these first archaeobotanical results from Bangladesh provide a baseline for developing further problem-oriented research on the origins and development of agriculture in Bangladesh.

### Geographical and archaeological setting

Bangladesh is a low-lying, riverine country located on the northern littoral of the Bay of Bengal. It is mostly formed of deep alluvial soils from the delta at the confluence of the Ganga, Brahmaputra and Meghna rivers and their tributaries. Bangladesh’s alluvial soils are highly fertile, but the low elevation and topography is prone to flooding (Alam et al. 2003; Allison et al. 2003; Kubo 1999; Rashid 2003). High monsoon rainfall (mostly between 1500 and 3000 mm per annum, but up to 5500 mm per annum along the northern border with Megalhaya, India) as well as a widespread of network river sources means that there is no shortage of water, and tropical forests flourish where they are not impacted by flooding or human disturbance. Elsewhere, riverine vegetation, tall perennial grasses and sedges are common, much as in parts of the Ganges plains (Meher-Homji 2001; Bhattacharyya 1997). The potential forest cover is generally classed with the moist deciduous tropical woodlands of eastern India, in which sal trees (*Shorea robusta*) together with *Cleistanthu*s are dominant. Towards the coast mangroves are prominent, and brackish wooded swamps may mark a transition to the freshwater zone, with palm trees such as *Nypa fruticans* and *Phoenix paludosa* as well as *Heritiera littorialis*, with tall buttressed trunks, much as in coastal tracks of Odisha and West Bengal (e.g. Puri et al. 1983, p. 413; Bannerjee and Ananda Rao 2001). Sal forests (mainly degraded) cover upland sites in the Madhupur tract. This forest probably covered all the upland areas before it was cleared for settlement and cultivation. Patches of edaphic grassland remained in the Madhupur Forest Reserve near Madhupur town at the time of the reconnaissance soil survey in the 1960s. Buried layers of organic matter accumulations in deep valleys near Dhaka are assumed to indicate that upper layers formed under reed swamp and the lowest layer under the mangrove forest (Brammer 2012), thus indicating the retreat of the coastline southwards as the alluvial delta of the Ganges-Brahmaputra-Meghna expanded through the Holocene. In addition to trees and shrubby elements, a number of common plants generally take a prominent role to provide a green cover with grasses or other herbaceous species throughout the delta region. Some important species are *Achyranthes aspera*, *Alternanthera sessilis* and *Blumea lacera*, although many of these may be secondary due to anthropogenic disturbance, as these taxa characterize “wastelands” and abandoned farm fields (Bannerjee and Ananda Rao 2001; Hossain 2008; Islam 2008).

Although human occupation is known to extend into the Lower Palaeolithic and Pleistocene, earlier Holocene prehistory is poorly understood. Lower Palaeolithic tools of the Anathyan prehistoric culture are similar to those from northeast India (Tripura, Assam) and Myanmar (Chakrabarti 2007, p. 3–4; Roy and Ahsan 2000, p. 21–32). However, sites from the early or middle Holocene that would be classed as Mesolithic, Neolithic or Chalcolithic are largely unknown, due no doubt to high levels of flooding and alluviation. Most of the prehistoric sites are found at the hilly region of the south-east and eastern part of the country. Prehistoric tools have been found but exact habitation context is unknown due to lack of excavations. In the hilly Madhupur and Barind tracts, highly fertile land for agricultural practices was available. Several Early Historic sites (starting in the last centuries BC or early centuries AD) are known from the Madhupur tract, such as Jangalbari (Zulkernine 2014). In the Barind tract, the best known Early Historic site is Mahasthangarh, although numerous sites of similar age are known in this area. However, their Neolithic or Chalcolithic precursors remain a mystery. Archaeological evidence becomes clearer in the Iron Age (ca. fifth to first century BC), connected culturally to the period of the Northern Black Polished Ware of Early Historic northern India (Rahman 2000). In this period large, urban sites appear in parts of Bangladesh, which are associated with place names known from ancient Indian textual sources (Chakrabarti 2000; Rajgor 2001; Chowdhury 2002). Wari-Bateshwar and Mahasthangarh were the main urban centres during the Early Historic period (500 BC–100 BC) (Ahmed 1979, 1981; Rahman 2000; Salles and Alam 2001; Haque et al. 2001; Pathan 1989; Rahman and Pathan 2013). While such sites can certainly be expected to have an agricultural base, the nature of that agricultural economy and what its local precursors might have been like is unknown. Thus, the current study aims to characterize the crop complex of this Early Historic agriculture, which provides a baseline from which to explore earlier origins. In addition, we have collected data from later sites (late 1st millennium AD), which provide some evidence on the longer term continuity or change in pre-modern agriculture in Bangladesh.

**Fig. 1:**
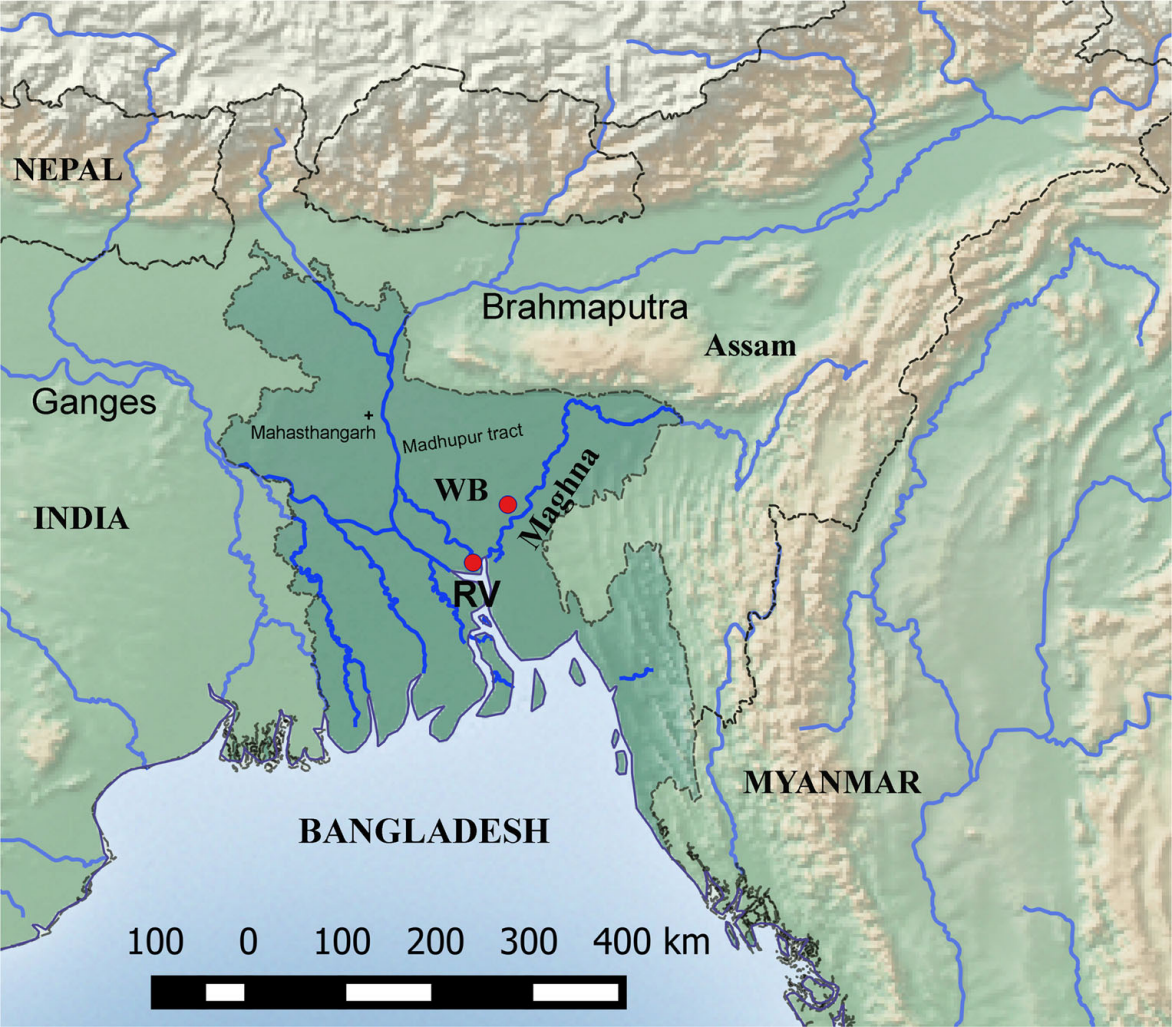
Map of Bangladesh and location of archaeological sites Wari-Bateshwar (WB) and Raghurampura Vikrampura (RV)

**Fig. 2:**
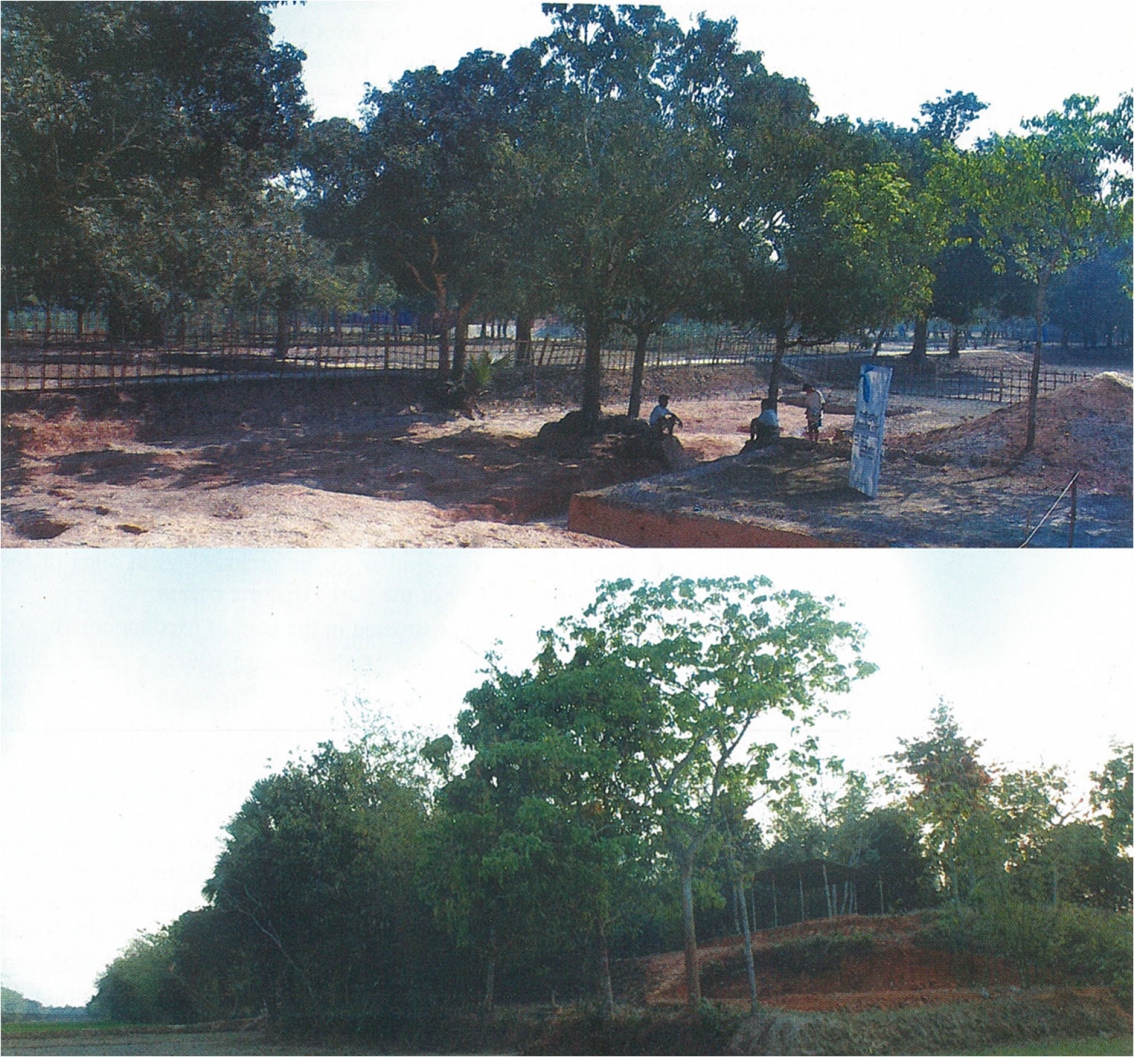
Photos of excavations of Wari-Bateshwar 2008 (above) and the edge of the site mound (below)

**Fig. 3:**
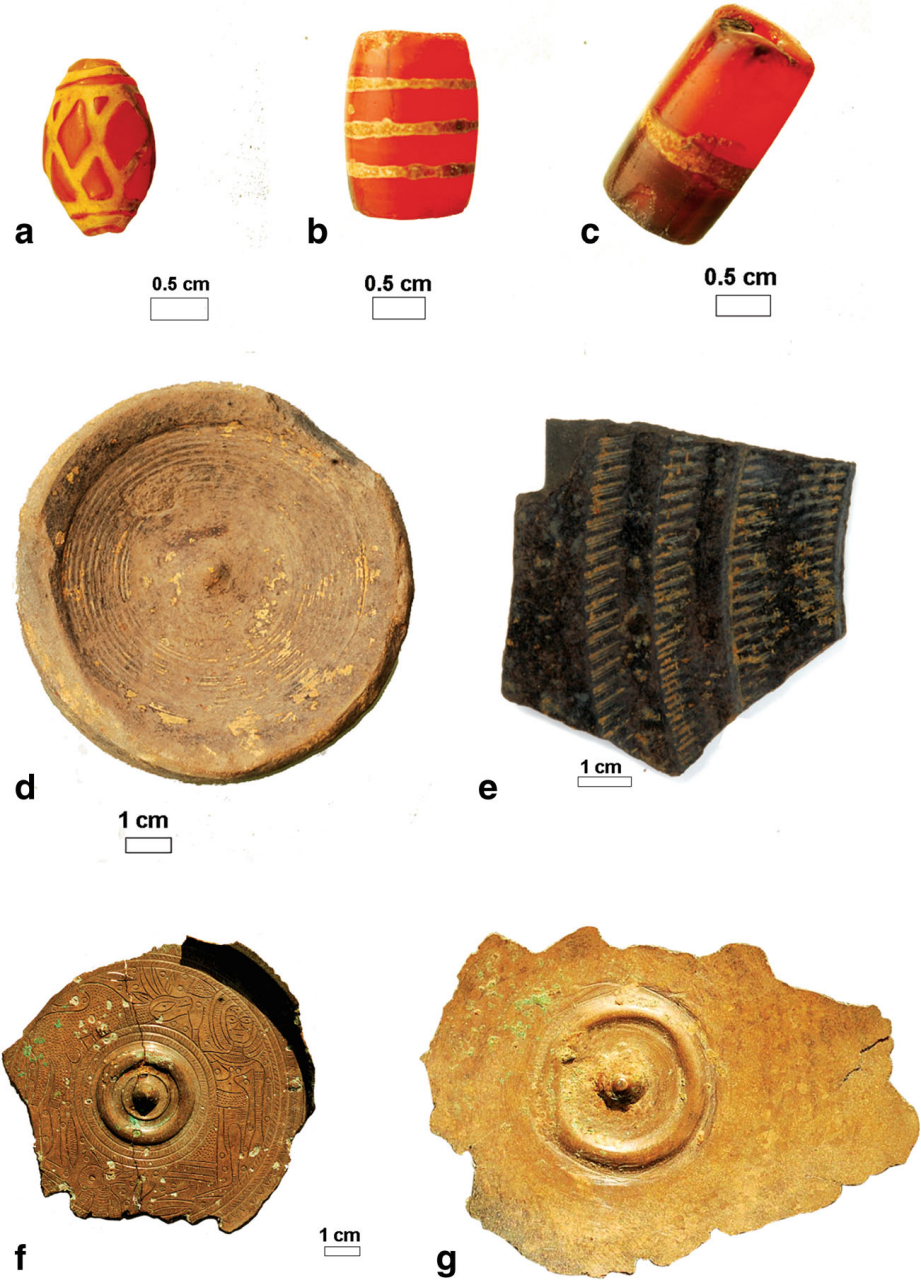
Examples of artefact finds from Wari-Bateshwar (500–300 BC). **a**, **b**, **c** Examples of etched carnelian beads. **d** Knobbed ware bowl. **e** Example of a Rouletted Ware sherd. **e** Incised copper vessel of type resembling local knobbed ware (from surface collection by Habibullah Pathan). **f** High-tin bronze frament of vessel resembling local knobbed ware

Wari-Bateshwar (WB) is an Early Historic archaeological site in Bangladesh, with apparent earlier levels dating back to the Chalcolithic (possibly 2nd millennium BC), but with its main occupation in the Iron Age (from the middle 1st millennium BC). It is located on the bank of the former Brahmaputra River channel in Narshingdi district (24° 05′ 34.6″ N, 90° 49′ 31.7″ E) (Fig. 1). This site has been known for finds of punch-marked coins since the 1930s (Chakrabarti 1992, p. 57; Rayhan 2011). The site has a citadel measuring 600 m^2^ with surrounding fortification and a moat. The river Koira flows to the north side of the site. Much of the former river channel is now silted up and under cultivation, apart from a very narrow canal, connected to the present Arial Kha River. The citadel is fortified by another fortification wall locally called *Asom Rajar Garh*, which is 5.75 km long, 5 m wide and 2 to 5 m high above the adjacent plain (Fig. 2). WB is also a find spot for Neolithic celts and shouldered axes of sand and fossil wood. From the main Early Historic levels (fourth–first century BC) find categories are numerous and include semi-precious stone beads, glass beads, punch-marked coins, iron axe and knives, copper bangles, a copper dagger, high-tin Bronze knobbed ware and ceramic knobbed ware (Fig. 3). Ceramic types including Northern Black Polished Ware, Rouletted ware, Knobbed Ware and Black Slipped Ware were recovered from WB and can be linked to North and South India and further trading connections with Southeast Asia (Basa and Rahman 1998; Bellina and Glover 2004). Brick structures, rammed floors and streets paved with potsherds are among the important features recorded. Radiocarbon dates place the main occupation in the fourth to first century BC, with some later construction of the site (Lotus temple) in the eighth to tenth century AD (Table 1). The discovery of a likely pit-dwelling in the deeper levels indicates the existence of an earlier, probably Chalcolithic settlement at WB (Rahman 2007, p. 29), speculated to date to the 2nd millennium BC (Rayhan 2011), although this remains undated and does not have associated archaeobotanical samples.

**Table 1:** AMS radiocarbon dates from Wari-Bateshwar and Vikrampura. Calibrated with OxCal 3.10 using the IntCal.13 curve

Lab number	Material [submission code]	Radiocarbon AGE	Calendar age (95.4% probability)	d13C
Wari-Bateshwar				
Beta-399412	Rice grain [WB-9]	2280 ± 30 BP	400–230 BC	− 24.7
Beta-401552	Rice grain [WB-4 SUPPLEMENT]	2290 ± 30 BP	400–255 BC	− 24.2
Beta-352967	Charcoal [WB Lotus Temple 1]	1190 ± 30 BP	AD 730–940	− 25.8
Beta-352969	Charcoal [WB structure 1]	2160 ± 30 BP	350–110 BC	− 26.4
Beta-352965	Charcoal [WB hearth 01]	2180 ± 30 BP	360–170 BC	− 25.3
Beta-399411	Rice grain [WB-4]	FAILED	–	–
Vikrampura				
Beta-401,554	Rice grain [RV2 SUPPLEMENT]	380 ± 30 BP	AD 1445–1630	N/A
Beta-401553	Rice grain [RV1 SUPPLEMENT]	520 ± 30 BP	AD 1330–1440	− 24
Beta-352963	Charcoal [VP-RRP2-1]	990 ± 30 BP	AD 990–1150	− 26.6
Beta-399413	Rice grain [RV1]	FAILED	–	–
Beta-399414	Rice grain [RV2]	FAILED	–	–

**Fig. 4:**
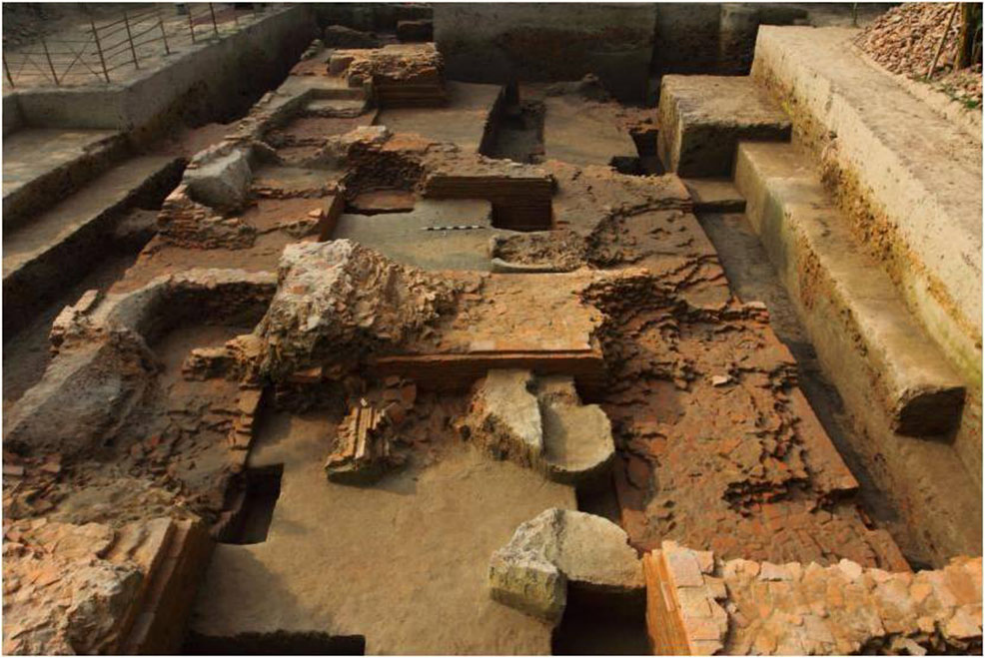
Photo of excavations at Raghurampura Vikrampura

Another archaeological site is Raghurampura (RV), located within the Vikrampura city site complex (23° 32′ 2.94″ N, 90° 29′ 24.0354″ E). It is located near the mighty Ganges (Padma) in Munshiganj District (Fig. 1). It was the location of a Buddhist Monastery (*vihara*). The site was excavated during the 2012–2013 and 2013–2014 seasons under the supervision of Sufi Mostafizur Rahman. Four rooms have been revealed within the *vihara* structure (Fig. 4). Thus far, samples were collected from the sediments lying immediately upon or composing the floor of this structure. Artefacts were dominated by pottery, bricks and brickbats. A radiocarbon date on wood (Beta-352963) could place the start of occupation in the eleventh or twelfth century AD, although the wood could be from an old tree. Direct radiocarbon dates on rice range from the fourteenth to early seventeenth century AD (Table 1). As the fourteenth century saw the conversion of much of Bangladesh to Islam, it could be that some or all of these plant remains date from a period of occupation or disturbance after the abandonment of the Buddhist monastic occupation.

## Material and methods

Sampling was carried out by simple bucket flotation, using the wash-over method. This method was pioneered by Helbaek (1969) and has been successfully applied to many sites in India (e.g. Fuller et al. 2004). In Bangladesh, the first excavations to carry out flotation were at WB and RV. At WB, sediments were collected from the fill of a trench feature excavated in 2009. This ancient trench measured 32 × 18 ft (approximately 6 by 8 m) at the top and is a reverse pyramid shape to a depth of almost 4 m. Its function is unknown, but it is presumed that its fill included secondary midden deposition from nearby occupation. The associated artefacts found indicate that it was abandoned during the Early Historic period. In addition, a figurine of a *yaksha* suggests art of the Indian Kushana period (first century BC). From WB, several layers from the Iron Age and Historic Period were sampled. In addition, at RV, all samples relate to the lowest levels of the excavated trench and are associated with fills above the floor level of the *vihara*; as indicated by the post-fourteenth century dates (i.e. Islamic period), these fills are likely to have been disturbed after abandonment of the monastic occupation.

As these were the first two instances of archaeobotanical sampling and flotation in Bangladesh, this was regarded as an experimental method. Although flotation may potentially affect carbonised remains through fragmentation, it is considered the most appropriate retrieval method for macro-remains in tropical environments. Dry-sieving would not be a good option as the mesh size would have to be larger than 250 μm (the mesh size used in the flotation process is 250 μm), and in the process, we would lose many smaller weedy taxa which provide information on ecologies. Flotation at WB took place in 2009 and at RV in 2013–2014 under the direction of the first author. The total number of samples and sample size was limited as the aim was to assess the potential of bucket flotation in these sites. At WB, we processed 16 samples, ranging from 4 to 10 l, a total of 98 l. At RV, we processed 7 soil samples totalling 52 l consisting, mostly of 2 l samples taken from the floor level, with one larger 40 l sample from underlying fill.

As there was no one in the country with experience in processing and sorting flots, the first author came to London in 2013 to receive laboratory supervision, to consult reference collections and to collaborate with the other authors (CM, CC, DF), who have experience working with archaeobotanical samples from South and Southeast Asia. Identifications were determined by experience and established identification criteria (by DF) as well as by comparison to the UCL archaeobotanical reference collection. For wild and weedy taxa, the likely family or genus is suggested as a thorough reference collection for Bangladesh or indeed the adjacent states of India is not available. Also a complete modern flora for Bangladesh was not available at the time of analysis, from which to assess plausible species present. All whole seeds and fragments were counted and included in the analysis.

## Results

**Fig. 5:**
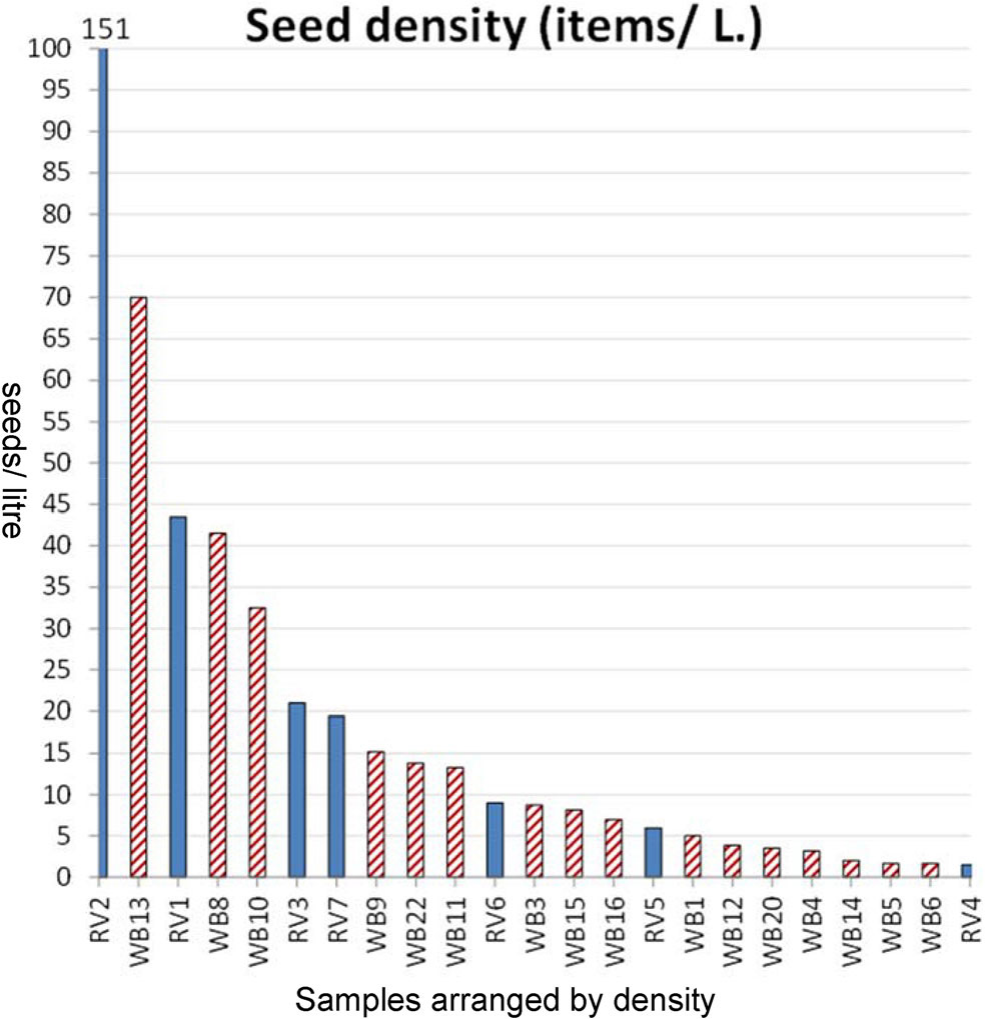
Density of seeds (and other non-wood charred remains) in the studied samples (items/litre)

**Fig. 6:**
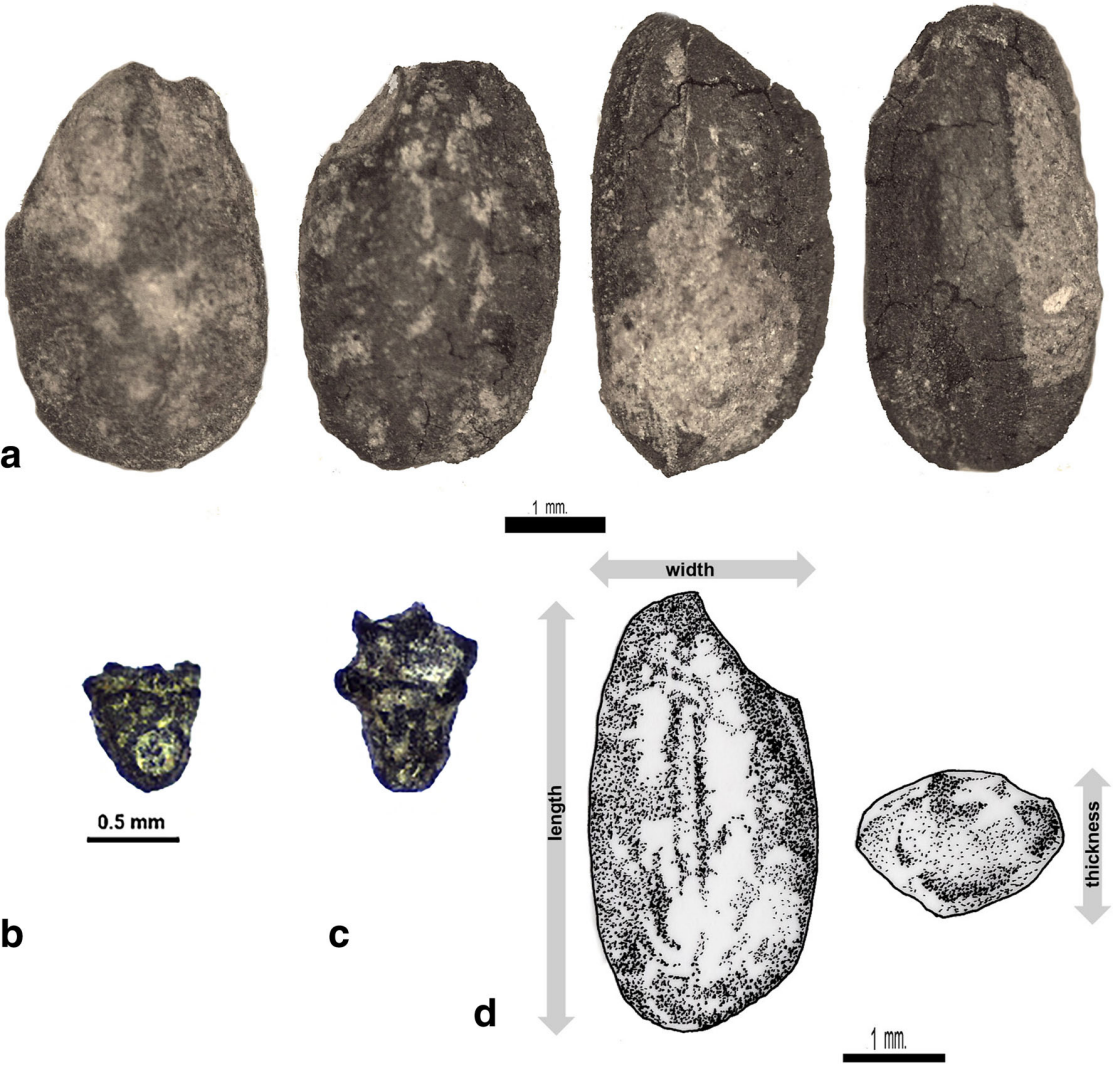
Examples of rice (*Oryza sativa*) remains from Wari-Bateshwar. **a** Charred grains. **b** Spikelet base seen from ventral (with scar) and dorsal view. **c** Spikelet based seen from dorsal view. **d** Drawing of a Wari-Bateshwar grain indicating measurements taken (by CC)

**Fig. 7:**
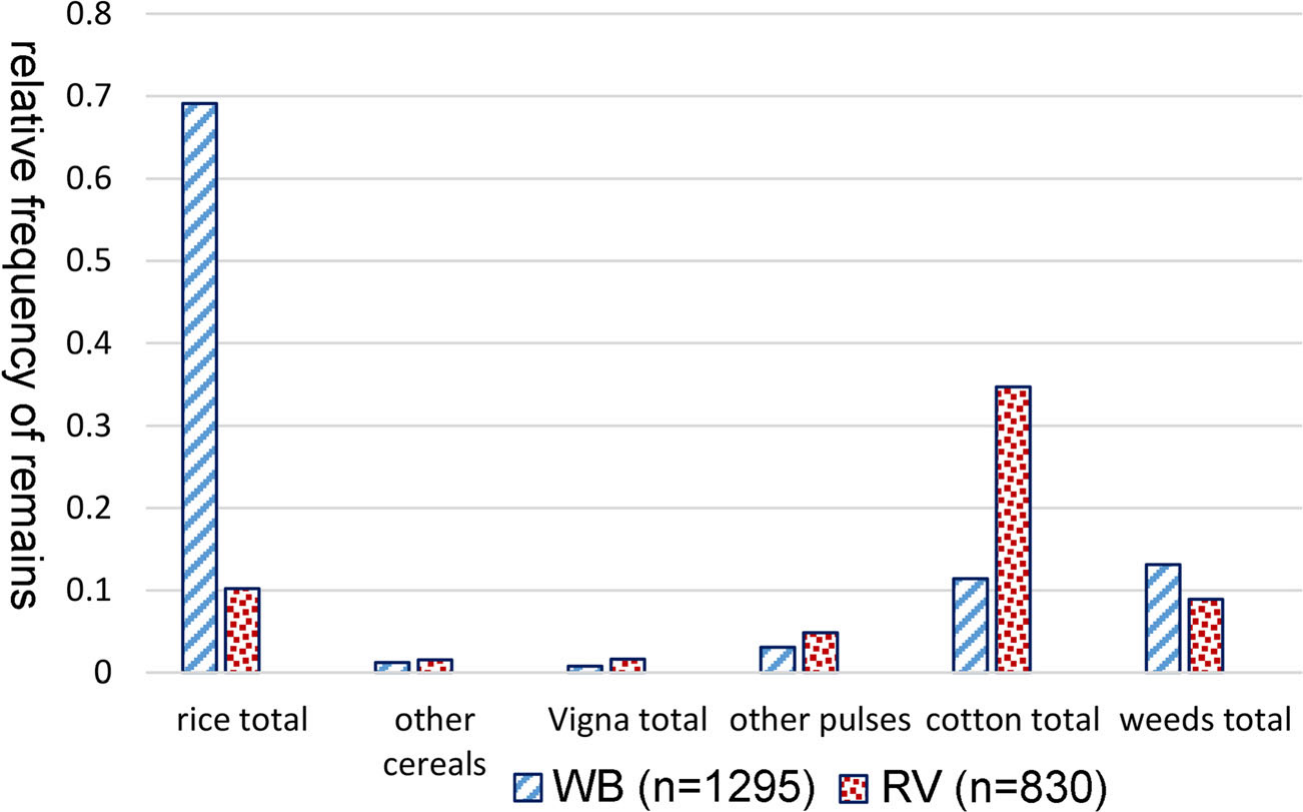
Relative frequency of major crop categories in **a** Wari-Bateshwar and **b** Raghurampura Vikrampura

**Fig. 8:**
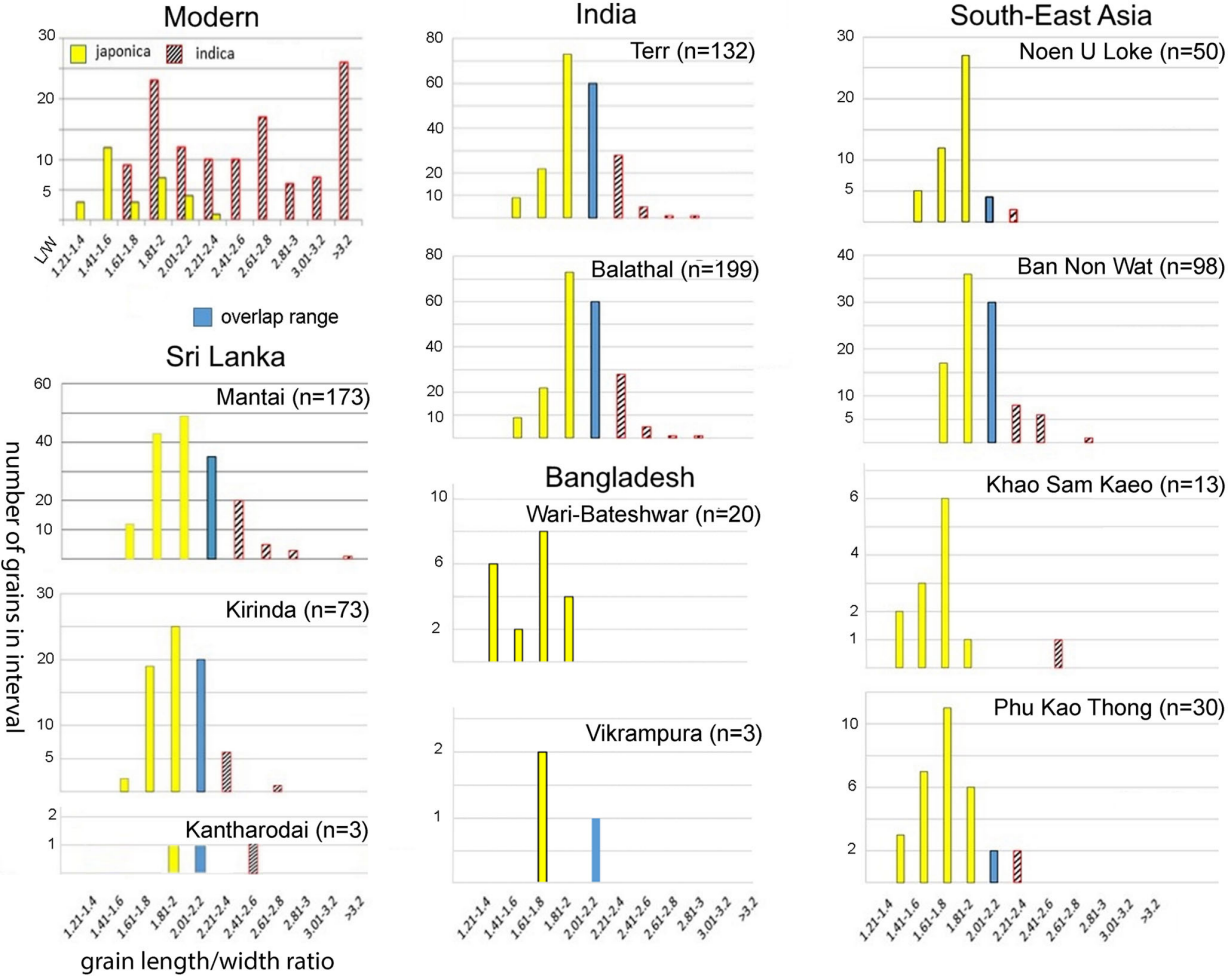
Rice grain length/width ratio distribution at Wari-Bateshwar and Raghurampura Vikrampura compared to other assemblages from South and Southeast Asia (all mature, charred grains), as well as modern reference material of subspecies *indica* and *japonica* (published assemblages and modern material from Castillo et al. 2016b; Murphy et al. 2018; Kingwell-Banham et al. 2018)

Despite the limited overall sample size, results were successful. At both WB and RV, ancient carbonized seeds and seed fragments were recovered. There were a total of 1295 botanical specimens at WB (Table S1) and 830 at RV (Table S2). Seed density averages were 14/L at WB and 23/L at RV, although the range was from as low as 1.5 items/L upto 151 items/L (Fig. 5). Forty-six distinct taxa were identified at WB and 45 at RV, including a wide diversity of pulses and weeds (Table 2; Table S1). The most common taxon at both sites was rice, represented by grains and spikelet bases with occasional lemma apex fragments and rachillae (Figs. 6 and 7). The second most common taxon was cotton (*Gossypium* cf. *arboreum*), largely encountered as seed fragments. Among the pulses a diversity of *Vigna* remains were found, indicating the presence of several taxa, predominantly the mung bean, but with rice beans (*Vigna umbellata*) at RV (Fig. 8). Both rice and *Vigna* spp. receive extra attention in the present paper.

**Table 2:** A summary of crop taxa present on the two sites, indicating counts of identifiable specimens. Full details in supplementary data

Taxon	WB	RV
Cereals		
*Avena sativa*	1	4
*Hordeum vulgare* rachis	1	
*Oryza sativa* grain/frag	162	98
*Oryza sativa* sipkelet bases	280	20
*Oryza* lemma apex	120	13
*Brachiaria ramosa/Setaria italica*		1
*Echinochloa* sp*.*	1	
*Pennisetum glaucum*	1	
*Setaria italica*		2
*Setaria* cf. *pumila*		1
*Setaria* cf. *verticillata*		2
cf. *Sorghum bicolor* (fragment)	1	
indet small millet	3	8
Pulses		
*Cajanus cajan*		1
*Cicir arietinum*		1
*Lathyrus sativus*	1	6
*Lens culinaris*	8	14
*Macrotyloma uniflorum*		2
*Pisum sativum*		6
*Vicia ervilia*		1
*Vicia faba*	2	5
*Vicia* cf. *sativa*		2
*Vicia* sp*.*	8	11
*Vigna* cf. *aconitifolia*		2
*Vigna* cf. *mungo*	1	3
*Vigna radiata*	11	5
*Vigna* cf. *trilobata*	2	3
*Vigna umbellata*		2
*Vigna* sp.	1	5
Other crops		
*Sesamum indicum*	3	3
*Gossypium* cf. *arboreum (whole)*	3	9
*Gossypium* cf. *arboreum (fragments)*	51	279
*Gossypium* cf. *arboreum funicular cap*	20	113
*Brassica* cf. *juncea*	1	2

Beyond rice (Fig. 7), other cereal evidence is limited, but includes a diversity of millets. Species level identification of millets is a challenge in South Asia, in part because so many taxa, native and introduced, can be counted among the millets (Fuller 2002; 2014), but useful criteria have been developed, as partially described in earlier works (Fuller 1999; 2003; Fuller et al. 2004; Murphy et al. 2018). The WB data included some evidence of larger-grained African millets, including one pearl millet (*Pennisetum glaucum*), and one probable *Sorghum (*cf. *sorghum bicolor*). A grain of *Echinochloa* sp. could be a weed of rice, although cultivated sawa millet (*E. frumentaceum*) cannot be ruled out. Other small millet fragments from WB could not be determined. By contrast at RV, no evidence of African millets were found, but two specimens of Chinese foxtail millet (*Setaria italica*) were identified. Separating *Setaria italica* from the South Indian native *Brachiaria ramosa* can be a challenge (see Fuller et al. 2004; Kingwell-Banham and Fuller 2014), and one specimen has been assigned to a *B. ramosa/S. italica* category. Two other foxtails, *Setaria* cf. *pumila* and *Setaria* cf. *verticillata*, were also present and may have been present as weeds of dry rice or millets, but their use as a grain cannot be ruled out given their importance to diet in other South Asian contexts (e.g. Weber 1991; Fuller et al. 2004). A diverse range of millets have turned up on Iron Age and Early Historic sites in the middle Ganges (Uttar Pradesh) (Fuller 2011), as well as in Chalcolithic Orissa (Harvey et al. 2006; Kingwell-Banham et al 2018a).

Among potential winter cereals, the absence of wheat is worth noting. A single specimen of barley comes from WB. To the West in the Ganges plains (Uttar Pradesh and Bihar) wheat and barley are nearly ubiquitous as crops, although never as frequent as rice, from around 2000 BC onwards (Saraswat 2004; Fuller 2011). Today, neither of these taxa is of much importance in traditional agriculture in Bangladesh nor further east into Southeast Asia, although both taxa are of importance further north in China. The finds of wheat and barley therefore help to outline the maximal extent of the early dispersal of the winter crops towards southeastern Asia. The few grains of oat from both sites may represent a weedy form (e.g. *Avena fatua*), as a weed in winter fallow fields where rice had grown or infestation of pulse crops, although some cultivation of oat (*Avena sativa*) is also possible. Oats have rarely been encountered archaeologically in South Asia (Figure S1). A report from Late Harappan Hulas (in western Uttar Pradesh) is noted but whether these specimens were a crop is unclear; a weed of wheat and barley seems more likely. In Kashmir, weedy *A. fatua* appears in the 1st millennium BC, while domesticated oats appear just at the end of the 1st millennium BC (Semthan period III) (Lone et al. 1993). Further east in Nepal domesticated oats have been found at the medieval (twelfth c. AD) site of Kohla in Nepal (Asouti and Fuller 2009), but in the central Nepal Jhong valley, they are absent from samples from the first Millennium BC through to the seventeenth/eighteenth century (Knörzer 2000). A single grain of oat is reported from Changgougou (near Lhasa), Tibet from ca. 1000 BC (Guedes et al. 2014). In general, cultivated oats are associated with cooler regions, such as in the Himalayas, so whether weedy or cultivated, the oats of ancient Bangladesh need further study.

### Rice: spikelet bases, morphometrics and variety

The dominant cereal in the samples is rice, suggesting that in the Early Historic and Medieval times, just as in more recent times, rice was the main staple grain in Bangladesh. In traditional agriculture in Bangladesh, one finds predominantly subspecies *indica* and *aus.* Long cycle, monsoon season rices, often irrigated, the so-called *aman* rice are predominantly subspecies *indica.* Two other traditional Bangladesh rices are in the circum-aus clade: these include shorter growth season, upland rices called *aus*, as well as winter irrigated rices, called *boro* (Hossain et al. 2012; Travis et al. 2015; see also Fuller and Castillo 2016: Table 1). Subspecies *japonica* appears to be rare today. There are also a few relict populations of wild rices (*O. rufipogon* and *O*. *nivara*) known from Bangladesh (Vaughan et al. 2008; Fuller et al. 2010). Therefore, identification of our archaeological rices needs to take into account any of these possibilities. Twenty rice grains from WB and three from RV were measured and used in the morphometric analysis. All rice plant parts including grains and spikelet bases are charred. The rice grains from WB are well preserved of which 18 are whole grains and two are fragments (Fig. 6a). Analysis of spikelet bases from these samples (e.g. Fig. 6b) indicates that the majority of these are domesticated (~ 83.4%) but with a large minority of wild type (~ 12.6%), and about 4% indeterminate or immature. This is consistent with domesticated rice populations which have a large presence of wild or weedy rices contaminating the fields (see, Fuller et al. 2016b). Similar percentages of wild rice are present in populations considered to have been domesticated rice in China, in both the Lower Yangtze (e.g. Liangzhu: Fuller et al. 2009) and in the Yellow river region (e.g. Fuller et al. 2010; Song et al. 2019), and wild rices can be expected to have been a prominent part of the natural wetlands of the Ganges-Brahmaputra-Maghna Delta.

Identified rice grains from WB and RV fall under two categories, either mature or immature. As a rice grain reaches maturity, length and width develop before thickness. Therefore, mature rice grains compared to immature ones are plumper, whereas immature rice grains, not having completely filled out are, normally of an average length and width, but thinner when the thickness is examined. Only mature grains were measured (Fig. 6c). There were high proportions of immature rice grains at WB and RV, 42% and 46% respectively, usually fragmented and these were not measured.

A simple morphometric analysis was used in order to identify the likely variety of rice found at WB and RV. Simple morphometric studies have been shown to be fairly accurate in differentiating subspecies in Asian rice studies, as backed by comparison with ancient DNA results from archaeological rice grains from India and Thailand (Castillo et al. 2016b). Charring causes distortions to rice grains normally resulting in shrinkage, particularly in the length of seeds (Bowman 1966; Braadbaart 2008). However, charring experiments conducted by Castillo (2018) showed that the width also decreased. This work indicated that amount of shrinkage for both the length and width is similar making the L/W ratio of charred remains comparable to those of modern rice measurements. Table 3 lists the rice grain measurements, while these are compared to previously studied and well-identified assemblages in Fig. 8.

**Table 3:** Rice measurements from two the sites Wari-Betashwar (WB) and Raghurampura (RV), including the length/width (L/W) ratio

Sample no.	L	W	T	L/W
WB-3	3.48	2.31	1.65	1.506
WB-12	4.02	2.64	2.07	1.523
WB-8	4.12	2.63	1.84	1.567
WB-9 (7)	4.19	2.67	2.18	1.569
WB-9 frag	4.28	2.71		1.579
WB-4	3.92	2.47	1.45	1.587
WB-9 (3) frag	3.87	2.32		1.668
WB-12	4.38	2.54	2.15	1.724
WB-9 (1)	4.59	2.5	2.27	1.836
WB-9 (5)	4.53	2.4	1.94	1.888
WB-11	4.41	2.32	1.55	1.901
WB-9 (10)	4.68	2.45	1.91	1.910
WB-9 (15)	4.78	2.49	2.08	1.920
WB-9 (6)	4.73	2.44	1.82	1.939
WB-9 (13)	4.54	2.34	2.15	1.940
WB-9 (2)	4.49	2.26	1.81	1.987
WB-9 (9)	4.49	2.25	1.78	1.996
WB-9 (4)	4.73	2.35	1.92	2.013
WB-9 (8)	4.05	2	1.6	2.025
WB-9 (11)	4.3	2.12	1.63	2.028
WB-8 frag		2.09	1.37	
WB-11 frag		2	1.42	
WB-4 frag		2.38	1.84	
VB-1	3.8	1.91	1.51	1.990
VB-2 frag	5.14	2.63		1.954
VB-2	4.87	2.08	1.84	2.341

The rice grains from WB showed variability in shape and size although the grains were relatively short and plump (Fig. 6a, c). The shape of *Oryza sativa* subspecies *japonica* is normally described as short and plump whereas *indica* rices are long and thin. The L/W ratios of the WB and RV rice grains were plotted against and compared to modern population measurements of domesticated rices of subspecies *japonica* and *indica* (Fig. 8). The L/W ratios of modern rice populations suggest that *japonica* normally falls below 2.01, whereas *indica* rice L/W ratios are above 2.2, with length width ratios between 2 and 2.2 constituting the major overlap zone between *japonica* and *indica.* Our preliminary modern data indicate that some *aus* rices, have L/W ratios greater than 2.81, comparable to many wild rices, although further comparative study across a wide range of *aus* varieties is needed. The morphometric analysis demonstrates that the L/W ratio of WB rice grains fall almost exclusively within the *japonica* range whereas RV is too small an assemblage to assign. We therefore suggest that the mature rice grains from WB are *japonica* type. This is of interest as this compares well with rice metrics from contemporaneous and earlier sites in mainland Southeast Asia, such as Ban Non Wat or Khao Sam Kaeo, but contrasts with contemporaneous assemblages in India (e.g. Balathal, Ter, Odisha Chalcolithic sites) and Sri Lanka (e.g. Kantharodai), which tend to indicate more *indica* dominant or mixed populations (see Fig. 8; Castillo et al. 2016b; Murphy et al. 2018; Naik et al. 2019). Early rice in Myanmar represented by impressions in bricks that may date back as early as the final centuries BC at Htaukmagon and Taungthaman also suggest shorter-grained *japonica* types (Watanabe and Tanaka 1981). The rice metrics therefore could be indicative of the diffusion of rice into Bangladesh from the east before the fourth century BC.

### Pulses

**Fig. 9:**
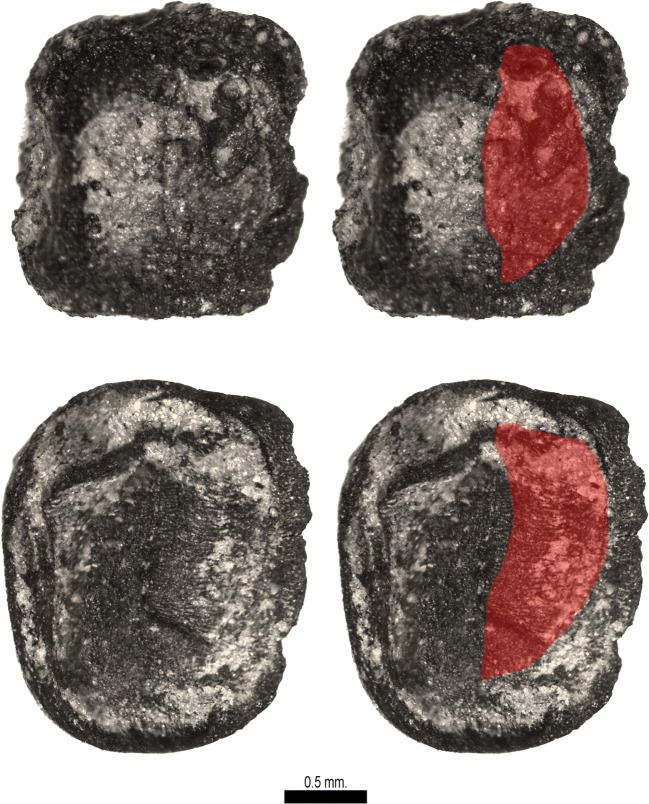
Photographs of two well-preserved examples of *Vigna radiata* from Raghurampura Vikrampura, showing the interior of the split cotyledon. At right, the same photos have the plumule indicated by shading

**Fig. 10:**
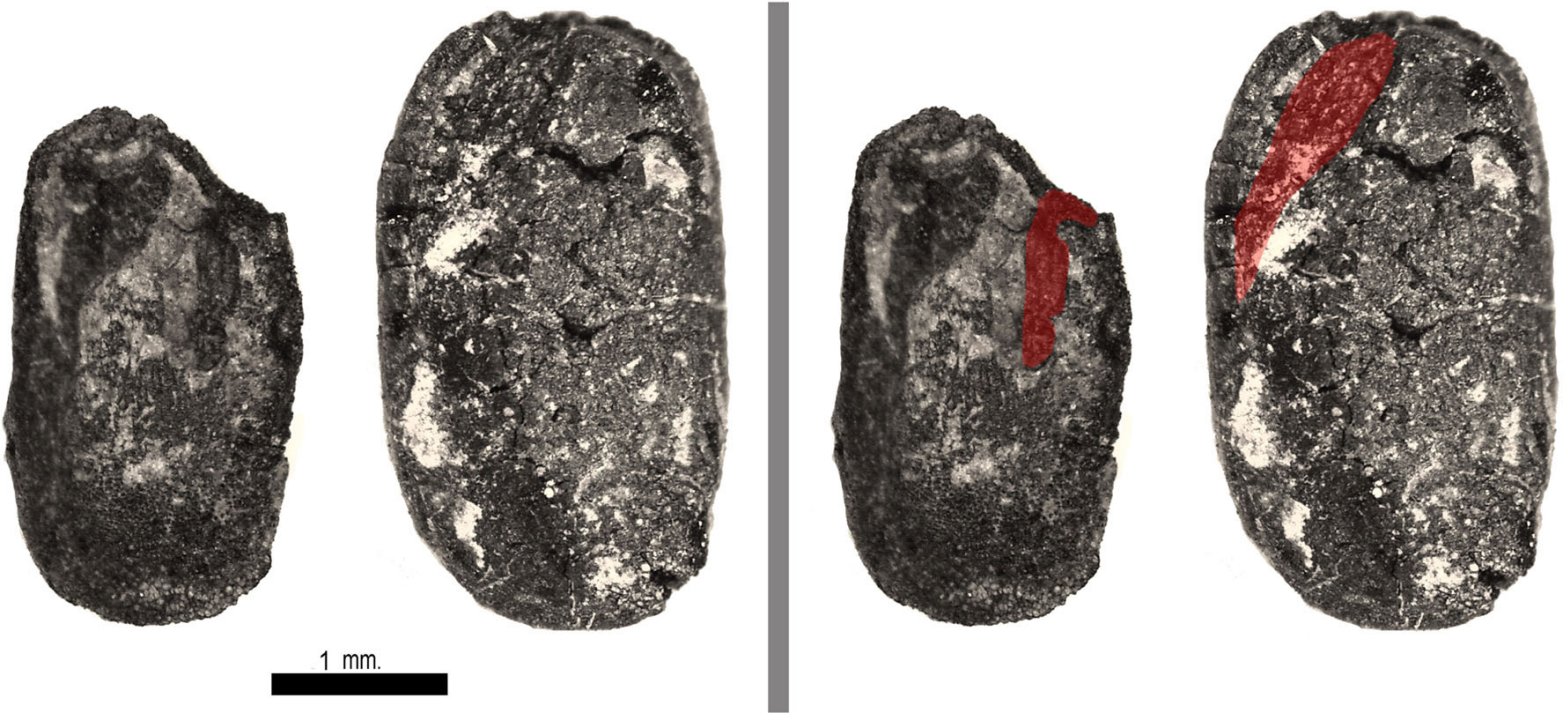
Photographs of two well-preserved examples of *Vigna umbellata* from Raghurampura Vikrampura, showing the interior of the split cotyledon. At right, the same photos have the plumule indicated by shading

Several tropical Asian pulses were recovered from both sites including black gram (*Vigna mungo*), mung bean (*V. radiata*), moth bean (*V. aconitifolia*) and horsegram (*Macrotyloma uniflorum*)*,* as well as some rice bean (*V. umbellata*), from RV. In addition, several pulses originally from the Near East were also recovered in low quantities including lentil (*Lens culinaris*)*,* common vetch (*Vicia sativa*), chickpea (*Cicer arietinum*) and pea (*Pisum sativum*)*.* The wide diversity of *Vigna* spp. makes this assemblage illustrative of the challenge of separating the *Vigna* crops. As described in Fuller and Harvey (2006), useful criteria include the ratio of the length of the plumule to the length of the seed, especially when the seedcoat and hilum are not preserved. This separates the longer plumule of *V. radiata* (Fig. 9) from the middling plumule of *V. mungo*, and the shorter plumule of *V. aconitifolia* and *V. umbellata.* Both *V. radiata* and *V. mungo* are wider (reflected in L:W ratio) than *V. aconitifolia* and *V. umbellata*. Moreover, the hilum of *V. radiata* and *V. mungo* are located near the middle whereas in the shorter plumule species, the hilum is off centre, with a larger and very off centre hilum in *V. umbellata* and a much smaller and only slightly off centre hilum in *V. aconitifolia.* While most of the *Vigna* spp., as well as *M. uniflorum* and pigeon pea (*Cajanus cajan*), are native to various parts of India and were all established in the Ganges Basin and probably Odisha by the later part of the 2nd millennium BC (Fuller and Harvey 2006; Fuller 2011; Fuller et al. 2019), *Vigna umbellata*, the rice bean, derives from wild populations in mainland Southeast Asia, either Myanmar or Thailand (Tomooka et al. 2003). Rice bean has previously been recovered from Iron Age southern Thailand (Castillo et al. 2016a). The specimens from RV, although not so well preserved as to be exemplary, still have characteristics that fit with this identification rather than other *Vigna* sp. Modern *V. umbellata* measurements range from 4 to 9 × 2.6–4 mm and have a short plumule usually spanning 40% of the length of the cotyledon (Castillo 2013). Two cotyledons from RV and their plumules were examined and measured (Fig. 10). The overall sizes of the two archaeological *V. umbellata* were adjusted for shrinkage by applying a 20% corrective factor and measured 3.6 × 2.1 mm and 4.4 × 2.6 mm. One specimen falls within the domesticated range whereas the other does not. However, it should be noted that both specimens from RV lack a testa which further decreases their size compared to the modern measurements which include the testa. The plumules of both specimens were ~ 40% the length of the cotyledons. The *V. umbellata* cotyledons from RV represent the first find in South Asia and suggest that this species spread into the Indian subcontinent by the eleventh century AD or earlier from the east. To date, the earliest and only archaeobotanical evidence comes from two sites in Southern Thailand towards the end of the 1st millennium BC when rice bean is found at Khao Sam Kaeo and Phu Khao Thong (Castillo 2013; Castillo et al. 2016a). Table 4 reports measurements on the well-preserved *Vigna* cotyledons from RV.

**Table 4:** Measurements on well-preserved *Vigna* pulses from RV

Sample	Taxon	Length	Width	Plumule length
RV-2	*Vigna* cf. *umbellata*	2.99	1.71	1.51
RV-10	*Vigna* cf. *umbellata*	3.63	2.17	
RV-1	*Vigna radiata* (w/ testa)	2.12	1.61	1.59
RV-2	*Vigna radiata* (no testa)	1.97	1.79	1.69
RV-2	*Vigna* cf. *mungo*	2.79	1.88	1.42

### Other crops

The category of ‘other crops’ represents the second largest category of the archaeobotanical assemblage, ~ 42% at RV and ~ 16% at WB. Other crops in the remains include oilseeds, fibre crops and one possible fruit tree. Of particularly note is the large presence of cotton (*Gossypium* sp.), mostly represented by seed fragments. While we do not know of a reliable method for distinguishing either the whole seeds or fragments of the two old world cottons (*G. arboreum* and *G. herbaceum*), we regard *G. arboreum* as more likely because it originated in Pakistan and was widely established across India in later prehistory (Fuller 2008), including Neolithic/Chalcolithic Hettapatti in the middle Ganges plains (Pokharia et al. 2017). In contrast, *G. herbaceum* must have been introduced from Africa to South Asia in pre-colonial times. Cotton seeds and fragments have been found in several sites in mainland Southeast Asia, the earliest one is from Southern Thailand dating to the Iron Age (Castillo et al. 2016a).

Two oil seed types are noted, a mustard seed (*Brassica* sp. or *Brassica* cf. *juncea*) and sesame (*Sesamum indicum*), both taxa that may have been brought into cultivation in the Indus valley region by the time of the Harappan civilization (Fuller and Madella 2002) and then spread through South Asia subsequently. Finds of these have generally been rare, probably due to the oily nature of the seeds, but both species are known from sites in the Ganges Valley, such as at Malhar (Tewari et al. 2000), Imlidh Kurd (Saraswat 1993a), Senuwar (Saraswat and Chanchala 1995), Narhan (Saraswat et al. 1994) and Raja-Nal-Ka-Tila (Pokharia et al. 2017). Sesame has also been found in Southern Thailand from the late 1st millennium BC (Castillo 2013; Castillo et al. 2016a) as well as fourteenth century Cambodia (Castillo et al. 2018a).

### Weeds

Numerous seeds of wild herbaceous taxa are present and these are regarded as probable weeds of cultivation. Given the dominance of rice most of these can be suggested to be weeds of rice, and therefore provide some insight into the nature of rice cultivation. However, reference material or manuals relevant to the identification of weed seeds in this region are not available, so most can only be assigned to likely family or sometimes genus identifications. Weed taxa that are suggested to be both from wet and dry ecologies occur on both sites, with the number of taxa and seeds of dry-cropping taxa outnumbering those of wet taxa (Tables S3-S4). The weed *Acmella paniculata* has been identified in WB and RV and is known to occur today in dryland cultivation systems across Southeast Asia (Moody 1989). *A. paniculata* has also been identified in archaeobotanical assemblages in Southeast Asian sites demonstrating rice farming systems were dryland during the prehistoric period (Castillo 2011; Castillo et al. 2016a, 2018b). And although it could be that some rice was grown under wetter field conditions, such as with irrigation, the evidence strongly suggests that fields were rainfed. It may also be that rice was grown under flood plain conditions, broadcast on uneven soils subjected to varying degrees of inundation by overbank flooding. The presence of some wetland weeds potentially indicate this (*Fimbristylis*, *Schoenoplectus*, *Ipomoea* and *Eleocharis*). We have observed such forms of cultivation with low field preparation investment in tribal areas of ethnographic Odisha (e.g. Field I7 in Weisskopf et al. 2014). This leads to the same fields hosting wet and dry weeds with wet weeds on the deeper water channel end of the field and dry weeds on other upper end. Such simple *decrue* systems may be low in productivity by modern standards, but would have required low labour investment and would have been potentially adequate to the needs of the first towns in Bangladesh. Larger sample sizes, higher resolution in taxonomic identification and phytolith analyses may help to clarify the nature of rice cultivation, and such work is currently ongoing.

## Discussion

### The sources of Bangladeshi crops

**Fig. 11:**
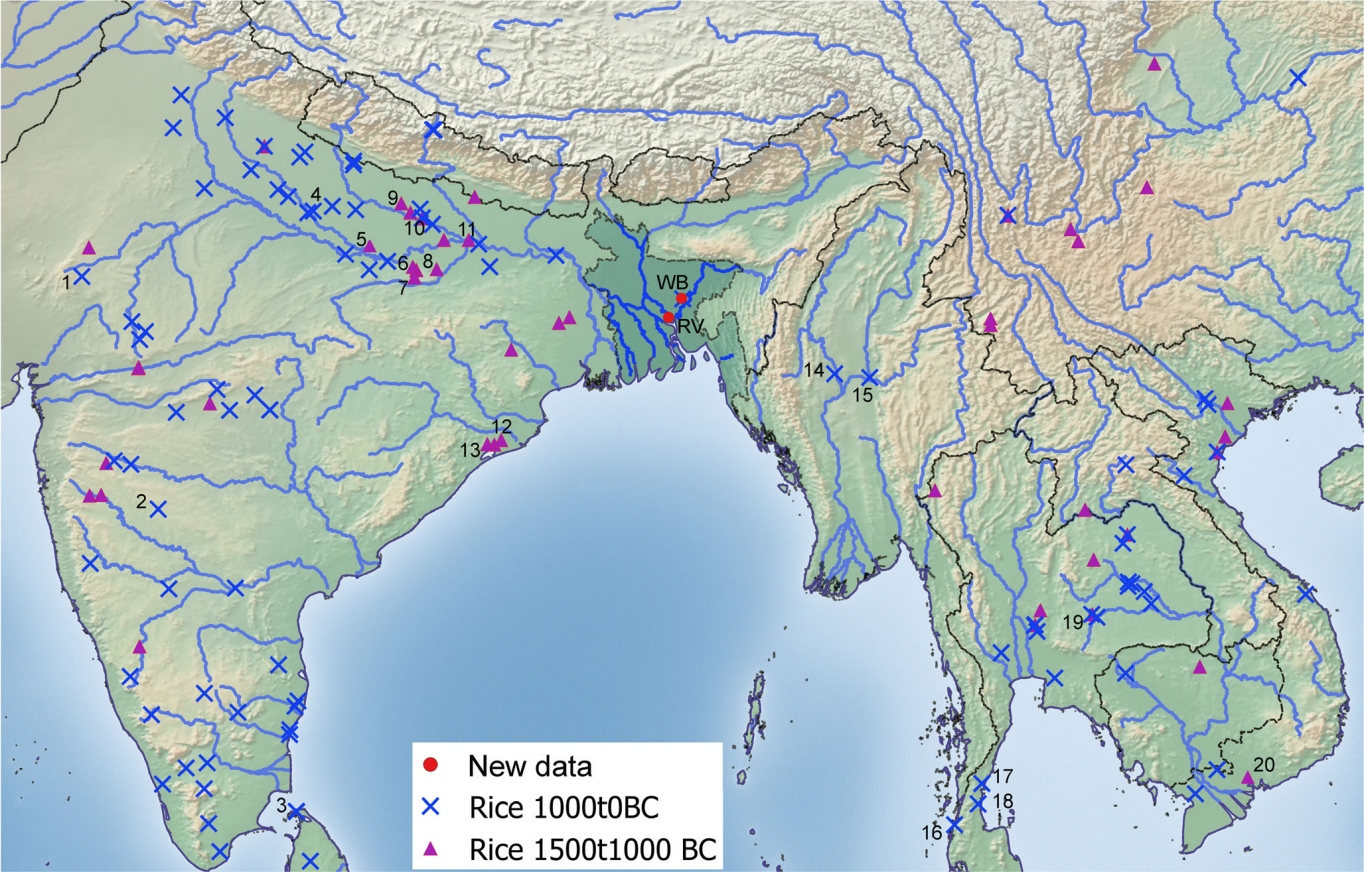
Regional archaeobotanical context of new data. This map plots the distribution archaeological rice finds from sites with median age between 1500–1000 BC and 1000–0 BC (data from Silva et al. 2015), selected sites numbered: (1) Balathal, (2) Ter, (3) Katharodai, (4) Hulaskhera, (5) Hettapatti, (6) Malhar, (7) Raja-Nal-Ka-Tila, (8) Senuwar, (9) Imlidh Kurd, (10) Narhan, (11) Chirand, (12) Golbai Sassan, (13) Gopalpur, (14) Htaukmagon, (15) Taungthaman (16) Phu Kao Thong, (17) Khao Sam Kaeo, (18) Khao Sek, (19) Ban Non Wot, (20) Rach Nui

Bangladesh falls between better documented archaeobotanical records of India and mainland Southeast Asia (Fig. 11). With regard to rice in particular, there are clear patterns in the establishment of rice in the 2nd millennium BC in northern-central India on the one hand and mainland Southeast Asia, e.g. Thailand, on the other hand, with the crops becoming more widespread in the Iron Age (Fig. 10); Silva et al. 2015). It remains unclear, however, when and by what routes rice became established in the intervening region represented by Bangladesh, northeastern India and Myanmar.

The current archaeobotanical results suggest diffusion into Bangladesh from both the west and east. The overall composition of the samples appears to have a character that is by-and-large South Asian. The dominance of rice and a mixture of summer and winter-grown pulses, is similar to Iron Age and Early Historic crop repertoires documented in the middle Ganges valley of northern India (Saraswat 2005; Fuller 2011; Pokharia et al. 2017). The diversity of millets, pulses and the presence of barley indicate differences from the more limited pulse diversity and rice focus of the Odisha region of eastern India (Harvey et al. 2006; Kingwell-Banham 2015). Sesame is also of Indian origin and presumably dispersed overland to Southeast Asia and China (Fuller 2003b) or via the Maritime Silk Route to the Peninsula of Thailand (Castillo et al. 2016a). The high occurrence of cotton suggests textile production was an important element of the economy by the Early Historic period. Although cotton is also known from mainland Southeast Asian finds by this period (e.g. Cameron 2010; Castillo 2013; Castillo and Fuller 2010; Castillo et al. 2016a), production in Southeast Asia was likely more limited, whereas major production took place in peninsular India (Fuller 2008). This cash-cropping and crafting industry also has its origins to the west of Bangladesh.

The presence of African millets is of particular note, although one hopes that additional evidence from future sampling will confirm their presence and importance. While both of these taxa arrived in western India by the Late Harappan era (2000–1700 BC) (Fuller 2003a; Fuller and Boivin 2009), only sorghum became established and important east of India, including as a major crop in China and parts of Southeast Asia. The presence of sorghum in Bangladesh near the end of the 1st millennium BC could mark the eastward journey of this crop. There are some dubious claims for Neolithic sorghum in China at 3000 BC (e.g. Li et al. 2010), which are not regarded as reliably identified (by DQF), and are notably absent from any professionally studied archaeobotanical assemblages collected by flotation. Written sources suggest that sorghum entered China from the Southwest, and through Sichuan in the first half of the 1st millennium AD (Lu and Dahlberg 2001). Pearl millet, by contrast, tends to be cultivated only in the driest parts of India (Harinarayana 1987), as it is originally a highly tolerant cereal of low rainfall conditions in Sahelian Africa (Tostain 1998; Manning and Fuller 2014). De Wet (1987) indicates that where rainfall is less than 800 mm pearl millet tends to dominate over sorghum. Pearl millet, however, is also salt tolerant (Harinarayana 1987), and there may be circumstances especially in more coastal or estuarine Bangladesh, where this trait was valued. Only two sites in the Ganges plains have produced clear evidence for pearl millet, chalcolithic Narhan and Imlidh-Khurd, both dating primarily to the 2nd millennium BC (Saraswat 1993a; Saraswat et al. 1994). A probable find also comes from Early Historic Hulaskhera (Chanchala 1992). These Gangetic sites and WB are all areas that are somewhat wet for expected significant cultivation of pearl millet, and suggest that cultivation in wetter zones was more common in the past than in recent times, and perhaps that some wetter-tolerant forms might have existed in the past. This has also been discussed in the context of pearl millet in Africa, since finds from the late 1st millennium BC from Cameroun fall outside the normal ecological range of modern landraces (Neumann et al. 2012; Kahlheber et al. 2014). This suggests also that pearl millet was found to be useful as a crop for some particularly stressed environments or else it was valued for diversifying the range of cereals and foodstuffs. Furthermore, millets can be counted among the minor crops grown on the drier margins of rice cultivation or perhaps intercropped with pulses.

Nevertheless, a couple of species point to likely Southeast Asian connections and the adoption of some crops from the east. These include the evidence for *japonica* type rice from WB, established already by the fourth century BC, as well as the later addition of rice bean at RV. In addition, among the weeds is the rainfed rice weed, *Acmella paniculata.* This species has proved to be nearly ubiquitous in archaeobotanical samples from Thailand, where it is suggested to be a major weed of rain-fed rice, as well as being used as a green vegetable (Castillo 2011, 2013, 2017b; Castillo et al. 2017). By contrast, this species has not yet been encountered in Indian archaeobotany. While it is possible that it was originally within the flora of Bangladesh, it seems more likely that it dispersed westwards from mainland Southeast Asia, presumably as a weedy contaminant of *japonica* rice. As such, it can be suggested that the rainfed rice cultivation practice diffused from the east into Iron Age Bangladesh, whereas other crops largely were adopted from India to the West. The presence of some wetland cultivation in Bangladesh may be down to the tendency for flooding to affect parts of the field rather than to the development of true wet-field, irrigated rice systems. Although wet rice systems are inferred to have become widespread in Gangetic India during the Iron Age (Fuller and Qin 2009), Iron Age agriculture in Thailand remains largely rainfed (Castillo 2017a). Therefore, despite a predominantly “Indian” character to the Early Historic crop and weed repertoire in Bangladesh we have indicators that Bangladesh was a crossroads with Southeast Asian traditions and had received some crop species and varieties via diffusion from the east.

The traditional rice diversity in Bangladesh raises a question that we remain unable to answer at present, namely the antiquity and origins of the *aus* varieties of rice. The material presented above fits with a provisional assignment of the rice largely to the *japonica* subspecies. As explored elsewhere *japonica* originated in China and diffused to early Southeast Asia (Fuller et al. 2010; Castillo 2011, 2017a; Castillo et al. 2016b), and at present, it is not clear when *indica* rices were introduced to mainland Southeast Asia, but likely no earlier than the 1st millennium AD (Castillo and Fuller 2010; Castillo et al. 2018b). India, however, while the original source of *indica* rice, also had received domesticated *japonica* rice from China by late Neolithic times (around 2000 BC or shortly thereafter) in order to provide a source for introgression of domestication genes from *japonica* into proto-*indica* (Fuller et al. 2010; Fuller 2011; Choi et al. 2017). Recent ancient DNA results from Early Historic rice grains from India confirm the presence of both *japonica* and *indica* subspecies, while contemporary grains from Thailand were entirely of the *japonica* subspecies (Castillo et al. 2016b)*.* Thus, with regard to the source of Bangladeshi rices, both India and mainland Southeast Asia could have supplied domesticated *japonica* rice to the early cultivators of Bangladesh, but the absence of *indica* grain metrics and the presence of the weed *Acmella paniculata* support rice diffusion from the east. This Early Historic evidence of *japonica* rice in turn implies that subsequent to this the *indica* and *aus* rices that dominate traditional Bangladeshi agriculture were introduced later.

The present results confirm the high potential for archaeobotanical research in Bangladesh. Even a most basic program of bucket flotation has produced both high diversity and relatively high seed density in archaeological sediments dating back 2000 to 2500 years BP. Further work can be expected to contribute to both outlining the early development of agricultural systems in Bangladesh as well as charting the movement of crops between the Indian subcontinent and Southeast Asia. Despite being a tropical country, with high rainfall and intensive seasonal soil processes due to the expansion and contraction of clays, our work proves that conventional archaeobotany, the collection of macro-remains through flotation, has much potential to put together a history of crops and agricultural systems in Bangladesh when sites have well-preserved stratigraphy. The first results here represent just a small step forward in a longer journey to better understand the spread and evolution of agricultural systems throughout South and Southeast Asia and the dynamics of crop introduction from west to east and east to west. We hope that archaeobotanical research can become a routine part of archaeological projects in Bangladesh, so that the archaeology of Bangladesh can become part of the larger story of the agricultural histories of Asia.

## Electronic supplementary material

ESM 1 (DOCX 613 kb)
