# Supplementary material for: Agricultural systems in Bangladesh: the first archaeobotanical results from Early Historic Wari-Bateshwar and Early Medieval Vikrampura
Source: Archaeol Anthropol Sci. 2020 Jan 15;12(1):37. doi: 10.1007/s12520-019-00991-5 (PMC6962288; doi:10.1007/s12520-019-00991-5)
Supplement: Supplementary file 1 — (DOCX 613 kb) [file 12520_2019_991_MOESM1_ESM.docx]

Figure S1 for” Agricultural systems in Bangladesh: the first archaeobotanical results from Early Historic Wari-Bateshwar and Early Medieval Vikrampura”


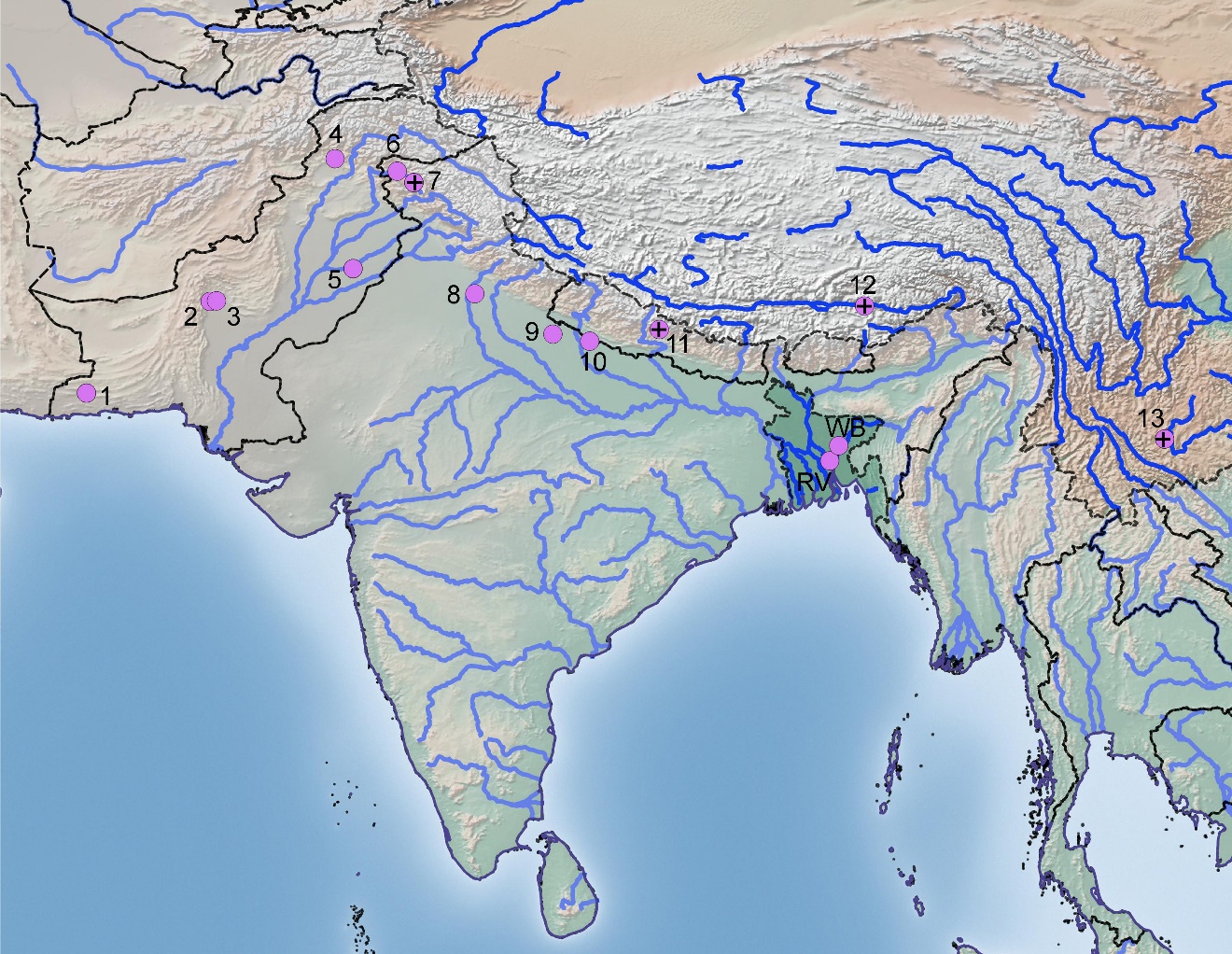


Figure S1. Distribution of archaeological oats (*Avena* sp.) in South Asia. Sites with black crosses represent domesticated crop remains, while others may represent weeds/ wild oats. Sites numbers and ages: 1. Miri Qalat (4000-2000 BC);-2. Mehrgarh V-VIII (3000-2000 BC) 3. Pirak (1950-1500 BC); 4. Bir-Kot-Ghwandai (1700-1400 BC); 5. Harappa (2600-1700 BC); 6. Qasim Bagh (2050-1675 BC); 7. Semthan (200 BC-AD 500); 8. Hulas (1800-1300 BC) 9. Saunphari (1000 BC-AD 300); 10. Charda (300 BC-AD 100); 11. Kohla ( ); 12. Kaerdong (450-700 AD); 13. Guanfentou (1500-700 BC). WB= Wari Bateshwar (400-100 BC); RV= Raghurampura Vikrampura (AD 990-1630).
